# Retailer Responses to Public Consultations on the Adoption of Takeaway Management Zones Around Schools: A Longitudinal Qualitative Analysis

**DOI:** 10.34172/ijhpm.8294

**Published:** 2024-08-27

**Authors:** Matthew Keeble, Michael Chang, Daniel Derbyshire, Martin White, Jean Adams, Ben Amies-Cull, Steven Cummins, Suzan Hassan, Bochu Liu, Antonieta Medina-Lara, Oliver Mytton, Tarra L. Penney, John Rahilly, Nina Rogers, Bea Savory, Annie Schiff, Richard Smith, Claire Thompson, Thomas Burgoine

**Affiliations:** ^1^MRC Epidemiology Unit, University of Cambridge School of Clinical Medicine, Cambridge, UK.; ^2^Department of Marketing, Faculty of Business and Economics, University of Antwerp, Antwerp, Belgium.; ^3^Office for Health Improvement and Disparities, Department of Health and Social Care, London, UK.; ^4^Department of Public Health and Sports Sciences, Faculty of Health and Life Sciences, University of Exeter, Exeter, UK.; ^5^Nuffield Department of Primary Care Health Sciences, University of Oxford, Oxford, UK.; ^6^Population Health Innovation Lab, Department of Public Health, Environments & Society, Faculty of Public Health & Policy, London School of Hygiene & Tropical Medicine, London, UK.; ^7^Department of Urban Planning, College of Architecture and Urban Planning, Tongji University, Shanghai, China.; ^8^Great Ormond Street Institute of Child Health, University College London, London, UK.; ^9^Global Food System and Policy Research, School of Global Health, Faculty of Health, York University, Toronto, ON, Canada.; ^10^School of Health and Social Work, University of Hertfordshire, Hatfield, UK.

**Keywords:** Childhood Obesity, Commercial Determinants of Health, Fast Food, Obesity, Thematic Analysis

## Abstract

**Background:**

Takeaway food is often high in calories and served in portion sizes that exceed public health recommendations for fat, salt and sugar. This food is widely accessible in the neighbourhood food environment. As of 2019, of all local authorities in England (n=325), 41 had adopted urban planning interventions that can allow them to manage the opening of new takeaway outlets in "takeaway management zones around schools" (known elsewhere as "exclusion zones"). Before adoption, local authorities undertake mandatory public consultation where responses objecting to proposals can be submitted. Evidence on common objections could be insightful for practitioners and policy-makers considering this intervention.

**Methods:**

We included 41 local authorities that adopted a takeaway management zone around schools between 2009 and 2019. We identified and analysed objections to proposals submitted by or on behalf of food retailers and local authority responses to these. We used reflexive thematic analysis with a commercial determinants of health lens to generate themes, and investigated if and how objections and responses changed over time.

**Results:**

We generated four themes: The role of takeaways in obesity, Takeaway management zone adoption, Use and interpretation of evidence, and managing external opinions. Despite not being implicated by the adoption of takeaway management zones around schools, planning consultants objected to proposals on behalf of transnational food retailers, however, independent takeaways did not respond. Objections attempted to determine the causes of poor diet and obesity, suggest alternative interventions to address them, undermine evidence justifying proposals, and influence perspectives about local authorities and their intervention. Objections consistently raised the same arguments, but over time became less explicit and expressed a willingness to partner with local authorities to develop alternative solutions.

**Conclusion:**

Objections to local authority proposals to adopt an urban planning intervention that can stop new takeaways opening near schools featured strategies used by other industries to delay or prevent population health intervention adoption. Practitioners and policy-makers can use our findings when developing proposals for new takeaway management zones around schools. By using knowledge about their local context and addressing arguments against specific aspects of the intervention, they can pre-empt common objections.

## Background

**Key Messages**

**Implications for policy makers**

- Our findings provide further evidence of a “playbook” used by multiple industries to object to population health intervention adoption. The arguments used across multiple industries are often the same, meaning that our findings are transferrable to other contexts.
- Planning consultants objected to local authority proposals to adopt takeaway management zones around schools on behalf of transnational food retailers. They used the same or similar text consistently over time. This consistency means that future objections can be pre-empted.
- Transnational food retailers would not be implicated by takeaway management zones around schools. Their objections may be an attempt to delay or prevent population health intervention adoption. National policy-makers need to be made aware of these potentially harmful food industry actions.

**Implications for the public**

Between 2009 and 2019, planning consultants working on behalf of transnational food retailers consistently objected to the adoption of “exclusion zones,” which can manage the number of new takeaways allowed to open near schools. Arguments used mirrored those used by the tobacco and alcohol industry when objecting to interventions aimed at them. For example, planning consultants claimed that there was little evidence to support takeaway management zone adoption, made poor diet and health seem like they were due to a single cause and suggested alternative interventions that would not stop the opening of new takeaways. “Exclusion zones” are intended to improve population health, especially among children. The findings from our research highlight the ways that large, recognisable, food retailers prioritise their future development and profits over the health of the next generation.

Neighbourhood food environments provide the opportunity to purchase food from multiple food retailers, including convenience stores, supermarkets, restaurants, and hot food takeaway outlets (“takeaways” hereafter).^1^ There is evidence that the opportunity to access food from such food retailers has increased in multiple countries since at least 2003, including in Australia,^2^ New Zealand,^3^ the Netherlands,^4^ the United States,^5^ and the United Kingdom.^6^ Focusing on takeaways, the food sold is often served ready-to-consume, intended to be consumed away from the point of purchase, and in portions that exceed public health recommendations for calories, fat, salt and sugar,^7-9^ and more frequent consumption has been associated with poorer diet quality,^10^ and living with obesity.^11^ As a result, population health interventions intended to reduce portion sizes and change the nutritional composition of takeaway food have been adopted and implemented.^12,13^ These interventions are intended to change individual-level food purchasing practices, but have mixed evidence of effectiveness.^13,14^

 An alternative approach is to limit potential exposure to takeaway outlets in the neighbourhood food environment. Doing so may offer health benefits because outlet exposure is proposed to precede the purchase and consumption of food sold.^15,16^ Urban planning interventions are one way that outlet exposure could be limited. Urban planning is referred to as zoning, city planning, and spatial planning in some countries and is a technical field. We therefore provide a glossary for the terms we refer to, applicable to England, in Table.

**Table:** Definitions for Planning Terminology Used Throughout Manuscript, in the Context of England

**Term Used**	**Description**
Adoption	The process of formally adopting a draft planning document, meaning that the policies and regulations it contains become a material consideration in deciding the outcome of planning applications.
Commercial determinants of health	Systems, practices, and pathways through which commercial industries influence health and equity.
Draft planning policy/regulation	A planning policy/regulation that is not yet a material consideration. Proposed policies/regulations published in draft Local Plan Documents are subject to examination by the National Planning Inspectorate.
Freedom of information request	A request for information held by a public authority, who have 20 working days to respond under the Freedom of Information Act 2000.
Harmful commodity industry	An industry that produces or sells harmful (ie, unhealthy) commodities such as sugar sweetened beverages, ultra-processed foods, or tobacco.
Local authority	An administrative body in local government that is officially responsible for public services and facilities in a given area.
Local Plan Documents	Documents that set out specifications and supporting justification text for planning policies/regulation, objectives, and targets that reflect the needs, concerns, priorities, vision, and objectives of a local authority.
Material consideration	A matter taken into account when deciding the outcome of a planning application.
National Planning Policy Framework	A set of national planning principles and policies that guide local authorities in developing Local Plan Documents and Supplementary Planning Documents.
National Planning Practice Guidance	National level guidance on how to apply the National Planning Policy Framework.
Planning policy	Controls the development and use of land, that can determine the appropriate use of a retail unit within the Use Class Order.
Public consultation	The process by which a local authority receives responses about draft Local Plan or Supplementary Planning Documents. Consultation responses can be broad or specific to a single element of proposals.
Public health responsibility deal	A voluntary commitment launched in 2011, where retailers could agree to change the nutritional composition of food sold (for example) as a way to promote improved population health.
Retailer	In the context of our study, food retailers who responded to local authority public consultations, regardless of food sold, their size (employees or outlet number), location, or classification within the Urban Planning system in England (eg, restaurants, takeaways).
Supplementary Planning Document	A document providing additional detail, context and justification to a policy contained in a Local Plan Document.
Takeaway management zones around schools	A designated area around schools where any new takeaway development could be managed.
Takeaways	Formally called “Hot Food Takeaways,” and in the context of urban planning regulations in England, defined as selling “hot food where consumption of that food is mostly undertaken off the premises.” Sometimes referred to as “fast-food outlets.” Hot Food Takeaways were categorised within the A5 use class until September 2020, after which, they were classed as “sui generis” (no specific use class).
The National Planning Inspectorate	Executive agency of the Department for Levelling Up, Housing and Communities of the United Kingdom Government that is responsible for examining draft Local Plan Documents and determining the outcome of appeals against planning decisions.
Transnational food retailer	Retailers (as defined above) operating in multiple countries and that are globally recognised in the fast-food retail sector. These retailers are often chains such as McDonald’s or Subway. In the UK, many of these retailers are *not* classed as “takeaways” or “hot food takeaways” in the context of urban planning.
Ultra-processed food	Ultra-processed foods are often industrially manufactured and include added fats and oils, free sugars and cosmetic additives including colours, stabilisers, humectants, emulsifiers that are not commonly used in domestic kitchens. These foods are typically hyper-palatable, affordable and convenient.
Use Class Order (pre-September 2020)	Classifications defined in national legislation, relating to the uses of buildings and retail units. A1: Shops (including the sale of food for immediate consumption on the premises) A2: Financial and professional services A3: Restaurants and cafes A4: Drinking establishments A5: Hot food takeaways

 In England, opening a new takeaway requires approval through a process of applying for planning permission, with decisions made on applications in accordance with a framework of policies.^17^ As of 2019, half of the local authorities in England (n = 325) had adopted a planning-based intervention that would enable them to prevent new takeaways from opening, with adoption starting from as early as 2009. Of these, 41 local authorities identified areas around schools where the opening of new takeaways would be managed to prevent future increases. Although these zones are sometimes known as “exclusion zones” we use “takeaway management zones around schools” or “takeaway management zones” to more accurately reflect the objective of the intervention. That is, not to necessarily exclude takeaways from operating or to close existing takeaways, but rather to prevent an increase in the number of *new* takeaways. In the context of these zones around schools, adoption partly reflects that childhood obesity rates in England have continued to rise since at least 2006,^18,19^ with over one-third of children in Year 6 of school (aged 10-11 years) identified as overweight or obese in 2021.^19^ Nevertheless, adoption would also plausibly reduce population-level takeaway exposure,^20-22^ meaning there are likely to be wider population health benefits. Despite this, planning and public health professionals in England with experience of attempting to have a takeaway management zone adopted in their local authority encountered barriers.^23,24^

 Before takeaway management zone adoption, local authorities in England undertake mandatory public consultation under the 2012 Town and Country Planning (Local Planning) Regulations for Local Plan Documents and Supplementary Planning Documents^25^ During public consultations, responses that support or object to proposals for a range of policies (ie, not only those related to takeaways) detailed within these documents can be submitted, with local authorities responding to outline if they agree or disagree, the rationale for their position, and if they have amended their proposal as a result (See Supplementary file 1: Figure S1 for an overview of this process). In public consultations held before the adoption of interventions targeted at tobacco, alcohol, and gambling industries, those likely to be most affected responded using approaches that attempted to delay or prevent the adoption of proposals, with strategies adapted over time based on previous experiences.^26-33^ These strategies have been recognised as part of a broader industry playbook, encapsulated as part of the commercial determinants of health, which recognises how industries influence societal norms and can influence the development of physical environments, including within the food retail sector. Given that the takeaway food industry may perceive takeaway management zone adoption as a threat to future development goals, they too may attempt to delay or prevent adoption. Nevertheless, the content of responses to takeaway management zone proposals and how they have changed over time have not yet been investigated. Better knowledge of this might help inform adoption and contribute to broader literature on attempts from harmful commodity industries to circumnavigate regulation.^34^ We therefore aimed to investigate whether and how takeaway food retailers objected to local authority proposals to adopt a takeaway management zone, how local authorities responded, and how these changed over time.

## Methods

###  Study Design

 We completed a longitudinal qualitative analysis of responses submitted during public consultations held by local authorities before the adoption of their Local Plan Documents or Supplementary Planning Documents. Ethical approval was not required.

###  Included Local Authorities

 We included local authorities in England that had adopted a takeaway management zone between January 2009 and December 2019 (n = 41, see Supplementary file 1: Box S1 for included local authorities). The timescale of our study was informed by our previous research,^35^ and our evaluation of takeaway management zone effectiveness.^36^

###  Identification of Public Consultation Responses

 Local authorities in England must publish summaries of responses received during mandatory public consultations for Local Plan Documents, and their own response to these.^25^ Summaries are available on local authority websites or upon request, and in some instances summaries are verbatim copies of responses received during mandatory consultation processes.

 Between January and May 2023, we used three approaches to identify and gather public consultation responses. First, we searched local authority websites, focusing on the “Planning,” “Local Plan,” “Development Plan,” “Consultation,” “Meetings,” and “Agendas” sections, using search terms including “Local Plan draft,” “Local Plan consultation,” “Supplementary Planning Document draft,” and “Hot food takeaway supplementary planning document.” Second, if our website searches were unsuccessful, we emailed local authority planning departments, or known contacts within a given local authority, and asked to be provided with public consultation responses. If we did not receive a response after five working days then we sent a second email request. Finally, if we did not receive a response to our second email request after five working days, we submitted a freedom of information request to the local authority.

###  Public Consultation Responses Included in Analysis

 Local authority public consultations are open for anyone to respond to. Responses can support or oppose proposals. Moreover, local authorities often propose broad and multi-faceted policies that can stop new takeaways from opening, for example due to non-conformity with litter or parking requirements. Given the aims of our study, we included responses from any food retailer (“retailers” hereafter) that objected to aspects of proposals specifically detailed as a takeaway management zone around schools (or an “exclusion zone”), plus any additional information about the responding retailer (we did not consider objections that referred to other elements of proposals). We also included the local authority response that corresponded to objections. We did not consider responses that supported local authority proposals or the urban planning classification of the retailers that objected to proposals, meaning that they could have been classified as fast-food outlets or restaurants in an urban planning context.

####  Public Consultation Response Extraction

 Researcher 1 (MK) led data identification and extraction. After extracting data for 22 local authorities (53%), MK met with Researcher 2 (MC) to discuss the data. MK and MC agreed that the data would allow our research aims and objectives to be investigated. Therefore, MK continued with data extraction for the remaining 19 local authorities. As we were investigating changes to objections and local authority responses over time, we organised data chronologically according to year of takeaway management zone adoption.

###  Data Analysis

 MK, MC, Researcher 3 (DD), and Researcher 4 (MW) were involved in all stages of data analysis.

####  Positionality Statement

 MK, MC, DD, and MW are part of a research team investigating takeaway management zones around schools from multiple perspectives, including; health modelling and economics. Across our research team, evidence generated from our research has supported and helped justify the introduction of population health interventions intended to limit exposure to takeaways in the neighbourhood food environment.

 Before analysis, MK, MC, DD, and MW individually reflected on their existing assumptions, positions as researchers and practitioners, and prior interactions with retailers in a research context. MK, MC, DD, and MW agreed that although there can be some benefits to takeaways opening, they are often outweighed by drawbacks, and that any prior interactions with retailers in a research context were not relevant to our current study. Collectively, the aforementioned researchers expected that objections would be similar to those from other harmful commodity industries meaning that elements of an “industry playbook,” including attempts to reframe the public health problem, shifting blame to other actors (including individuals) and suggesting non-solutions would be evident in the data.^37-40^ This expectation informed our use of a commercial determinants of health theoretical lens to interpret retailer objections.^41-44^ Although we recognise the starting point of our analysis, given that our study was the first to investigate retailer objections to takeaway management zone adoption, we also ensured that we did not constrain our analysis or interpretations to only existing concepts.^45^

####  Longitudinal Thematic Analysis

 MK led data analysis, with support from MC, DD, and MW. Data analysis consisted of longitudinal thematic analysis that followed the principles of reflexive and codebook thematic analysis outlined by Braun and Clarke.^46-48^ We considered this to be appropriate because we acknowledged that some codes would be informed by our commercial determinants of health theoretical lens, and that our assumptions and previous experiences would contribute to theme generation and interpretation.

 In practice, MK used a deductive and inductive approach to code the data.^49^ Deductive coding was informed by previous research investigating responses to public consultations and the commercial determinants of health.^41-44^ Inductive coding involved consideration of uncaptured concepts.^49^ Using this hybrid approach meant that we started analysis with existing concepts but could add codes where appropriate. It also allowed us to describe retailer objections that were explicit in the data and interpret their meaning. MK generated initial themes from the coded data and evaluated changes over time. Although we expected retailer objections to change over the period of our data (2009-2019), we did not develop an a priori timeline of events.

 MK shared a theme and code map (See Supplementary file 1: Table S1), example data, and interpretations with MC, DD, and MW. In two virtual data workshops, and communication via email in the intervening period, MK, MC, DD, and MW discussed the generated themes and interpretations, and reflected on any differences in opinion. The remaining authors contributed to theme interpretation during manuscript preparation. In line with Braun and Clarke,^46-48^ themes should be interpreted as representing shared patterns of meaning across the data.

## Results

###  Data

 We identified retailer objections to takeaway management zone adoption for 32 local authorities (78% of sample). For the nine local authorities (22%) with no data, our website searches indicated that retailers had not objected to proposals. This was confirmed through email communications or Freedom of Information requests.

 Separate planning consultants were commissioned to object to public consultations on behalf of separate transnational food retailers (McDonald’s Corporation, Kentucky Fried Chicken Corporation, and Domino’s Pizza, Inc.). Planning consultants explicitly named the retailer that they were responding on behalf of. At least one planning consultant objected to almost all local authority proposals, with only a small number of examples where no planning consultant objected. Although submitted by planning consultants, we refer to these objections as being from retailers because they had commissioned professionals to object on their behalf. Despite the opportunity to do so, local or independent takeaways did not respond to public consultations, and one industry representative association (The Pizza, Pasta & Italian Food Association) responded in 2010 but not again.

 Next, we provide an overview of retailer objections and local authority responses. This is followed by the findings of the themes we generated during analysis.

###  Retailer Strategy

 Retailers recycled the same text and arguments in their objections to local authority proposals. Reflective of this, retailer objections were consistent over time and did not vary according to the local authority, specifications of the proposal, or if proposals were detailed in Local Plan Documents or Supplementary Planning Documents. Despite primarily using the same core arguments, there was a subtle and nuanced change over time in the rhetoric of objections, including acknowledgement that the diet and health of young people and the broader population needed to be improved.

###  Themes

 We generated four themes: The role of takeaways in obesity, Takeaway management zone adoption, Use and interpretation of evidence, and managing external opinions. The data that contributed to these themes overlapped, which reflects that retailers used multiple, simultaneous, arguments, and strategies to object to proposals. We present verbatim quotes to illustrate each theme.

####  The Role of Takeaways in Obesity

 Retailers downplayed the contribution of their foods to excess energy consumption and obesity. Instead, they emphasised that children had access to energy-dense and nutrient-poor food from multiple different places. In referring to other sources of food, retailers appeared to shift blame for poor diet and obesity away from themselves and the takeaway food industry as a whole.

 “*[…] food of high energy density or poor nutritional value is sold from and at a range of premises within a variety of other classes, including many in Class A1, such as coffee or sandwich shops, bakeries or, simply, supermarkets, and that focusing on Class A5 uses is both unhelpful and unfair”* (Retailer: 2019).

 Highlighting other food sources alluded to the complexity of food access and obesity. Not explicitly referring to complexity allowed retailers to suggest alternative solutions that focused on single elements of diet and health (which we discuss further in the theme “Takeaway management zone adoption”). In contrast, local authorities explicitly recognised the complexity of food access and obesity and stated that multiple departments had contributed to the development of their proposal. This difference between retailers and local authorities underscores that the term ‘complexity’ can have multiple uses.

 “*The Council has not suggested that preventing an over concentration of A5 uses near schools is the only solution to addressing concerns over child health in the borough. Rather it is part of a multidisciplinary partnership approach across Council services”* (Local authority: 2015).

 Over time, retailers acknowledged that some (other) takeaways sell energy-dense and nutrient-poor food. Consistent with their attempts to evoke the complexity of the food system, retailers suggested that they should not be grouped with others because their food was different.

 “*Some hot food takeaways, together with restaurants, pubs and shops are clearly a source of cheap, energy dense and nutrient poor foods, however, not all hot food takeaways, restaurants, pubs and shops are, and the planning system is ineffective in distinguishing between those that are and those that are not*” (Retailer: 2015).

 Retailers also suggested that individuals should take responsibility for their food choices and health, and that diet variety and exercise, over which they had no influence, had not been considered by local authorities. Additionally, retailers placed the responsibility of managing the food intake of children onto parents.

 “*The inclusion of primary schools is particularly problematic as it is clear that children at primary schools are not usually permitted to leave the premises at lunchtime and, given their age, are unlikely to travel to or from school unaccompanied. Outside school time, children’s diets are quite properly the responsibility their parents or guardians”* (Retailer: 2013).

 A focus on individual responsibility may have been a purposeful attempt by retailers to further distance themselves from what they outlined as the “true” causes of poor diet and obesity.

 “*[…] the concept of “unhealthy eating” is unhelpful if isolated from consideration of wider issues such as diet variety and activity levels”* (Retailer: 2016).

####  Takeaway Management Zone Adoption

 Having declared what they considered the causes of poor diet and obesity, retailers suggested how they could be addressed. This did not include takeaway management zone adoption or wider attempts to prevent new takeaways from opening. Instead, retailers suggested that local authorities should promote physical activity. Creating a narrative of individualism allowed retailers to advocate for the adoption of market-based downstream interventions, which they argued were appropriate and aligned with their existing attempts to improve population health. This emphasis helped portray diet as a secondary obesity prevention consideration.

 “*In spatial terms, we consider there is a clearer link between obesity and lack of access to open space, sport and recreation, which could be positively promoted through the Core Strategy. Focussing planning resources towards this instead would ultimately be more productive in providing rather than removing choices on healthy lifestyles and would bring forward rather than delay useful policy”* (Retailer: 2009).

 Moving on to takeaway management zone specifications, retailers questioned the way that local authorities had determined the size, shape and anchor point of their proposed zones and challenged the inclusion of certain locations and retail classifications but not others. Doing so served to introduce doubt about the justification for, and appropriateness and effectiveness of proposals. This framing seems non-coincidental since the National Planning Inspectorate assesses proposals in Local Plan Documents based on them being justified and effective.

 “*Accessibility analysis based on “as the crow flies” radii, rather than real walking routes always overstates the area accessible in a certain walk time. […] Whilst we agree with approaches based on limiting over-concentrations generally, no justification is given for assessing this based on distances from schools”* (Retailer: 2015).

 “*Consideration needs to be given to the urban form as 400m as the crow flies is different to walking 400m. For example a train line could separate a site from a school meaning that the walking distance would be much further than as the crow flies”* (Retailer: 2018).

 During public consultations held closer to 2009, retailers explicitly objected to takeaway management zone adoption. Over time, retailers seemed to view adoption as somewhat inevitable, and so changed the presentation of their objections. Although explicit objection to takeaway management zone adoption became less apparent, retailers added underhandedly critical comments through text in brackets. Doing so reinforced their view that local authority proposals should not be adopted.

 “*Notwithstanding our objection to the principle of the policy, the distance of 400m from a school requires clarification and justification. Given this distance is quoted as a walking distance, this should be from the principle school entrance (if at all) and should not include playing fields or the like”* (Retailer: 2015).

 Between 2009 and 2017, retailers argued that takeaway management zone proposals from local authorities did not conform to the National Planning Policy Framework because this did not refer explicitly to diet, health, or takeaways. Referring to these zones as not being “feasible,” “viable,” “acceptable,” or “sustainable” further undermined proposals. In response, local authorities argued that the National Planning Policy Framework discussed healthy communities, and therefore, dietary health and obesity prevalence could be considered. When the National Planning Policy Framework was updated to refer to food environments between 2017 and 2018, retailers adapted their objections. Unlike before, they focused on how proposals did not support or promote sustainable development, and economic growth. Shifting focus in this manner indicates that retailers had an “object no matter what” attitude.

 Retailers stated that takeaway management zones would lead to a loss of employment opportunities, wages, and economic prosperity through business rate payments. However, these unintended consequences could be avoided through non-adoption.

 “*Because no assessment has been made of the number of hot food takeaways that might be refused as a result of this [takeaway management zone adoption] or what the social, economic or environmental impacts of that might be, it is not possible to balance these impacts”* (Retailer: 2015).

 Rather than indicating how they themselves would be affected, retailers displayed a concern for smaller takeaways and the local authority community. In an urban-planning context, the retailers objecting to proposals are classified as restaurants rather than takeaways and so they would not be directly implicated by takeaway management zone adoption. Making objections seem like they were on behalf of others indicates that retailers viewed themselves at the top of a takeaway food industry hierarchy.

 In response and contrast, local authorities framed takeaway management zones positively because of an ambition to protect the health of children whilst creating conditions supportive of economic prosperity.

 “*The final policy approach is considerably less restrictive than the moratoriums imposed by some other local authorities and is thought to provide an appropriate balance between striving to protect the health of children and enabling new businesses to become established”* (Local authority: 2014).

####  Use and Interpretation of Evidence

 Retailers and local authorities framed and interpreted the same evidence from academic research differently, in a manner that supported them. Doing so added apparent credibility to their respective objections and responses.

 “*[research] found that just 3/10 purchases were at A5 takeaways. 70% of purchases within the 400m school fringe were at A1 or A3 units, and concluded ‘the most popular shop was the supermarket, with more visits than all takeaways put together’” *(Retailer: 2014).

 “*Three out of ten purchases were made in takeaways and were generally for hot food such as chips, chicken or pizza”* (Local authority: 2009).

 Retailers stressed that evidence of an association between takeaway exposure and poor diet and health was weak because it was from cross-sectional and observational research. Local authority responses recognised that any conclusions drawn from research with these study designs should include caveats, but that the rationale for adoption should not be downplayed.

 “*There is a lack of evidence to demonstrate the link between fast food, school proximity, and obesity. This has been confirmed by Public Health England and the Local Government Association. Their paper, ‘Healthy People, Healthy Places’ states there is ‘an unavoidable lack of evidence that can demonstrate a causal link’ between fast food, school proximity and obesity. The same paper states there are only ‘theoretical arguments for the value of restricting the growth in fast food outlets’”* (Retailer: 2016).

 Despite their criticisms of the evidence from academic research, retailers used it to support their objections. When doing so, they used deterministic, authoritative and assertive language. Even when presenting evidence that did not demonstrate causality, retailers used terms such as “it is clear that.” This contradiction highlights an asymmetry in which retailers used evidence to support their position yet criticised local authorities for doing the same.

 Over time, more local authorities successfully adopted takeaway management zones around schools. Those local authorities holding public consultations emphasised this success.

 “*As of January 2017, there were 40 local authorities in England with policies or draft policies designed to restrict hot food takeaways in their local areas. One of the most common policies within these was that of Exclusion Zones around schools. It is important to use other councils as a guide to see how effective the documents are in achieving their goals”* (Local authority: 2018).

 Retailers ignored this trend and provided examples of local authorities that had not been successful. Retailers also depicted local authorities as being incompetent because even when a takeaway management zone had been adopted, it could not be implemented. The evidence regarding implementation was, however, somewhat redundant as public consultations were not concerned with this.

 “*Consequently, it is far from clear how refusing planning permission for hot food takeaways “close to” primary schools could ever be justified. This was the view taken by a Planning Inspector in an appeal (APP/P4415/A/11/2159082) against refusal of a restaurant and hot food takeaway in January 2012”* (Retailer: 2017).

####  Managing External Opinions

 Retailers consistently attempted to manage how their brands and reputations were perceived by the public and the National Planning Inspectorate. We summarise this under the term “healthwashing.” Similar to corporate social responsibility activities, retailers emphasised that they provided calorie information on menus, offered a variety of food choices to their customers, and funded sports in schools. Moreover, retailers portrayed themselves as victims when referring to how they were being “outcast” and “marginalised” and questioned why they were being isolated from other sources of energy-dense and nutrient-poor food (See theme: The role of takeaways in obesity). Doing so helped frame local authority proposals as being “unfair” and “unjust,” and a “moratorium” and “embargo” on takeaways.

 “*As a responsible business, McDonald’s recognises it has a role to play to support its staff, customers, and the communities in which it operates to live healthier lifestyles. For this reason, McDonald’s has invested significantly to evolve its menu over the last 10 years – both to extend the range of choice, and to reformulate our products”* (Retailer: 2014).

 Retailers criticised takeaway management zones as being restrictive rather than preventative, and outlined that adoption would negatively impact personal freedom. Doing so helped to create an illusion that local authorities represented the nanny state who stopped individuals from making volitional food purchases. On the other hand, local authorities presented themselves as “protectors” of young people. Furthermore, local authorities showed willingness to be flexible to retailer objections by amending their proposals, for example, by not including primary schools or changing the distance used in calculations. Although this may have been an attempt to secure adoption, it is possible that it embedded an unequal power dynamic and strengthened expectations that local authorities compromise and succumb to retailer demands. Nevertheless, there was one instance where a local authority exposed the repetitiveness of objections from one retailer.

 “*[…] responses were very similar to the response to Islington’s supplementary planning document, especially in terms of the evidence base documents cited. The respondent also provided comments on the draft Southwark Local Plan in March 2015 which are very similar to comments provided here. While there is no requirement for the respondent to have a bespoke response for different plans in different boroughs, these responses spanned a period from October 2013 to September 2015. In this time, the respondent has not added any additional evidence to reinforce their opposition to policies restricting A5 uses near schools”* (Local authority: 2016).

 Finally, retailer objections contradicted commitments to improving population health that they had made elsewhere. Attempting to prevent adoption does not correspond to being part of the Public Health Responsibility Deal. This apparent incongruity underscores how transnational fast-food retailers operate across the entirety of the food system, rather than in silos, and that their self-portrayal does not match their actions.

 “*Kentucky Fried Chicken (Great Britain) Limited is committed to working in partnership with government to increase the availability of healthy diet and exercise choices. It has delivered on this by signing up to the Department for Health Responsibility Deal […]”* (Retailer: 2016).

## Discussion

###  Summary of Findings

 We analysed public consultation responses from between 2009-2019 to investigate retailer objections to the adoption of takeaway management zones around schools, local authority responses to these objections, and changes in both over time. Planning consultants submitted objections on behalf of transnational fast-food retailers, with formulaic and almost identical text used consistently over time. We generated four themes through longitudinal thematic analysis with a commercial determinants of health theoretical lens: The role of takeaways in obesity, takeaway management zone adoption, use and interpretation of evidence, and managing external opinions. Retailer objections primarily attempted to ensure that local authority proposals were not adopted. Their objections featured strategies that undermined the possible effectiveness of this intervention, and attempted to divert attention to other sources of energy-dense and nutrient-poor food. Simultaneously, objections proposed alternative interventions that failed to acknowledge the complexity of food access and obesity and instead focused on individuals and their non-food-related behaviours. For example, improving levels of physical activity was suggested as a plausible alternative. There was little change to retailer objections over time. Although the core content was consistent, there was a subtle shift in how objections were ordered, and a desire to partner with local authorities to improve population health was introduced. In their responses to retailer objections, local authorities defended their proposal and the protection of their local area and community. To ensure takeaway management zone adoption, local authorities showed that they would amend their proposal in an apparent compromise to retailer demands.

###  Strengths and Limitations

 Our analysis of data from between 2009 and 2019 allowed us to investigate how retailer objections and local authority responses changed over time. This is likely to offer greater insight than data from a single time point. By analysing publicly available data, we were able to consider the nature of retailer objections when they were in the open. This provides alternative insight to non-public objections, since those in the open can influence public attitudes.^50^ However, we analysed published objections. We did not include private communications between retailers and local authorities that possibly occurred, meaning that their potential influence on takeaway management zone adoption is unknown. This limitation is not unique to our research.^43^ Additionally, local authorities did not always publish retailer responses verbatim, which limited our analysis in some instances. We addressed this by cross-referencing summarised objections with verbatim ones when they were from the same retailer. Moreover, objections were typically submitted by planning consultants on behalf of transnational retailers. Although retailers might have framed their objections differently in some instances, their use of planning consultants implies that they wanted objections to be professionally constructed and contextual to urban planning in England.

 We included local authorities in England with an adopted takeaway management zone. However, we did not include local authorities that may have proposed this intervention and subsequently did not adopt it, and we are unable to comment on retailer objections that may have been submitted after 2019. It is plausible that any objections we did not analyse and those from after 2019 were different. However, given that objections submitted between 2009 and 2019 consistently featured the same core content and repetition of the same text and arguments, it seems likely that later objections would have been similar.

 Our analysis and interpretations were informed by previous evidence on the commercial determinants of health.^41-44^ The policy dystopia model,^40^ or framing theory,^51^ would have plausibly provided alternative insight. During analysis, we did not determine themes *a priori* as this may have introduced bias, and we used inductive coding alongside deductive coding, which allowed us to consider interpretations that did not necessarily fit our analytic lens. Furthermore, multiple researchers were involved in data analysis and discussed interpretations in multiple meetings, which enhances the credibility and dependability of our findings.^52,53^

###  What Our Study Adds to Knowledge

 A selection of transnational food retailers (or planning consultants on their behalf) objected to local authority proposals to adopt a takeaway management zone around schools between 2009 and 2019. Although these objections are permitted, the retailers that responded would not necessarily be impacted by adoption because, in the context of urban planning, they are not classified as takeaways. As such, objecting to proposals appears to be a direct attempt to interfere with the adoption of a population health intervention that could plausibly contribute to obesity prevention. Using planning consultants implies that transnational food retailers are sufficiently concerned about risks to their immediate and future commercial interests that they want to ensure that their objections correspond with the nuances of the planning system in England. In contrast, independent takeaways did not object to local authority proposals. Independent takeaway owners may believe that objecting would be redundant because their size means they have no political leverage, or they may have a lack of knowledge about how to object. Moreover, independent takeaway owners are plausibly less likely to want to open new outlets in the future so may see no need to object. Given that existing takeaways are not forced to close due to takeaway management zone adoption, a complementary population health intervention would be to change the portion size and nutritional composition of food sold. Interventions of this nature were found to be feasible and acceptable to multiple stakeholders in England.^54,55^

 Retailer objections referred to different sources of energy-dense and nutrient-poor food, such as supermarkets, despite these not being included in proposals and without recognising that they also sell a variety of non-ready-to-consume food items. In doing so, objections made implicit arguments about complexity, which may have created uncertainty about theoretical and feasible links between consumption of the food that retailers sell and poor diet and obesity. It is possible that this narrative helped to promote that it would be redundant to intervene on a single source of food when there is an abundance of access elsewhere in the food environment.^56^ Creating doubt and referring to complexity are strategies consistently used by alcohol, tobacco, and other food and beverage industries when attempting to prevent or delay the adoption of population health interventions specific to them.^43,44,57,58^ To the best of our knowledge, objections to takeaway management zone adoption had not previously been investigated. The similarities we observed suggest that the novel findings from our study have relevance beyond the immediate context of takeaway food. Moreover, they contribute to a growing understanding of a cross-industry “playbook” that is dynamic and subject to amendment to suit contextual needs, even when interventions are proposed at a local rather than national level.^30,42,59-61^

 Over the ten-year period of our data (2009-2019), there was a subtle and nuanced shift in the nature of retailer objections. Retailers had the opportunity to incorporate experience developed over time, and to cite research from an evolving evidence base. However, there was a lack of wholesale changes to objections. In some ways, this suggests that the motivation of retailer objections is to ensure that their political and lobbying power is maintained and that they are present in policy-making processes.^34,62^

 Retailers and local authorities have different roles in the public consultation process. Retailers appear to be in a position of power whereby they can suggest changes to proposed interventions to make them better aligned with their corporate and economic priorities. On the other hand, local authorities are expected to amend proposals and continually justify their position. The role of the food industry in public consultations and policy development has been documented elsewhere,^31,32^ with industry power recognised as a constraint on obesity prevention measure adoption.^63,64^ Rhetoric around local authorities representing the nanny-state can particularly influence public perceptions and support for population health intervention adoption,^65^ which helps explain why this narrative featured throughout retailer objections. The impact of this rhetoric remains unclear, but could be investigated by examining if and how local authorities amended their proposals in light of retailer objections.

 Retailers and local authorities used the same evidence from academic research to support their respective agenda, yet framed their reporting of it differently. Moreover, retailers argued that there is a lack of evidence to support local authority proposals, which is another element of the industry playbook.^59,66,67^ Although retailer objections were based on the citation of a limited number of academic publications, the evidence base that justifies takeaway management zone adoption can be seen as limited and equivocal. For example, two studies based on data from England showed contrasting results in terms of the number of new takeaways opened following takeaway management zone adoption.^68,69^ However, these studies were limited to small areas that are unlikely to reflect the full extent of nationwide variation in takeaway management zone effectiveness. A national evaluation estimated a decrease in the number of applications received by local authorities with an adopted takeaway management zone.^36^ In turn, this suggests that the number of new takeaways would not have necessarily increased. It will be interesting to monitor how retailers amend their objections as the evidence base evolves.

###  Policy and Practice Implications

 Planning consultants commissioned by transnational food retailers explicitly objected to takeaway management zone adoption and consistently used arguments that mirrored those from harmful commodity industries objecting to population health interventions specific to them. Practitioners and policy-makers considering the proposal of this intervention can use our findings to anticipate objections they will receive during mandatory consultations and circumnavigate them before they are used. For example, we identified that ambiguous definitions such as “unhealthy eating establishment” was a point of dispute. Local authorities can prevent the future use of this argument by being providing rationale for their definition. This might involve the use of local context to provide clarification on apparent minutiae of their proposals. Furthermore, as it stands, academic literature has used language that potentially reinforces retailer self-portrayals as victims, which in turn might prevent adoption.^56^ Local authorities could amend how they frame these interventions to ensure that it does not benefit or support retailer narratives. For instance, “exclusion” could be replaced with a term that is neutral and favours a positive health narrative whilst also clarifying the intent of the interventions. That is, to manage the development of new takeaways in the food environment. We have started this evolution in terminology by referring to local authority interventions as being “takeaway management zones around schools.”

 The number of local authorities with adopted takeaway management zones around schools increased between 2009 and 2019.^35^ Although it appears that a precedent for adoption has been set, there are examples of local authorities failing to adopt proposals or having to amend them to ensure adoption.^70^ Local authorities considering this intervention might base their proposals on those already in place with minor adaptations to reflect their local need. Doing so might make the adoption process easier with less scope for challenge. Moreover, either in their proposals, local authorities might wish to provide a list of other local authorities with already adopted takeaway management zones as supporting evidence (See Supplementary file 1: Box S1). In turn, this would promote the normalcy and acceptability of this intervention.

###  Unanswered Questions and Future Research

 Practitioners and policy-makers in England acknowledge the importance of academic research evidence in the adoption of planning-based population health interventions.^23,24^ Nevertheless, it has been argued that the existing evidence base regarding takeaway exposure and health outcomes is limited and insufficient to support intervention adoption.^24,71^ Retailers and local authorities selectively cited academic research evidence to support their respective positions. Future research that evaluates the quality of the evidence cited and the accuracy of reporting might help to understand criticisms of existing research and inform future research priorities.

 The findings of our study are based on objections submitted on behalf of a limited number of transnational food retailers. These retailers have the resources to use planning consultants to identify and respond to local authority public consultations. However, their objections do not necessarily reflect the entirety of the food industry. Understanding if and how perspectives about takeaway management zone adoption differ among independent takeaways would be informative, as they may be affected differently.

 Finally, the experiences of local authorities that proposed a takeaway management zone but subsequently did not proceed with adoption are unclear. Based on our findings, if retailers objected to adoption, it is likely that they used similar arguments. Why some local authorities do not proceed with adoption if they receive objections could be particularly important to understand both in the context of our research and the adoption of other population health interventions.

## Conclusions

 Between 2009 and 2019, planning consultants commissioned by transnational fast-food retailers explicitly objected to proposals to stop new takeaways opening near schools in takeaway management zones. The arguments and strategies used were similar to those from other harmful commodity industries, including attempts to undermine evidence justifying adoption, shape narratives about the causes of poor diet and obesity, propose alternative interventions to address these, and influence public perceptions about local authority intentions. We anticipate that planning consultants will continue to object to takeaway management zone adoption on behalf of transnational fast-food retailers. Our findings will allow local authorities considering adoption of this population health intervention to pre-empt common objections they may receive.

## Ethical issues

 Ethical approval was not required for this research because no human participants were involved. All data used in analysis were publicly available.

## Conflict of interests

 Authors declare that they have no competing interests.

## Supplementary files

Supplementary file 1 contains Table S1, Figure S1, and Box S1.
