# Supplementary material for: Retailer Responses to Public Consultations on the Adoption of Takeaway Management Zones Around Schools: A Longitudinal Qualitative Analysis
Source: Int J Health Policy Manag. 2024 Aug 27;13:8294. doi: 10.34172/ijhpm.8294 (PMC11496767; doi:10.34172/ijhpm.8294)
Supplement: Supplementary file 1 — contains Table S1, Figure S1, and Box S1. [file ijhpm-13-8294-s001.pdf]

Article title: Retailer Responses to Public Consultations on the Adoption of Takeaway Management Zones Around Schools: A Longitudinal Qualitative Analysis

Journal name: International Journal of Health Policy and Management (IJHPM)

Authors' information: Matthew Keeble[1,2]*, Michael Chang[3], Daniel Derbyshire[4], Martin White[1], Jean Adams[1], Ben Amies-Cull[5], Steven Cummins[6], Suzan Hassan[6], Bochu Liu[1,7], Antonieta Medina-Lara[4], Oliver Mytton[8,1], Tarra. L Penney[9], John Rahilly[8,1], Nina Rogers[1], Bea Savory[6], Annie Schiff[1], Richard Smith[4], Claire Thompson[10], Thomas Burgoine[1]

1. MRC Epidemiology Unit, University of Cambridge School of Clinical Medicine, Cambridge, UK.
2. Department of Marketing, Faculty of Business and Economics, University of Antwerp, Antwerp, Belgium.
3. Office for Health Improvement and Disparities, Department of Health and Social Care, London, UK.
4. Department of Public Health and Sports Sciences, Faculty of Health and Life Sciences, University of Exeter, Exeter, UK.
5. Nuffield Department of Primary Care Health Sciences, University of Oxford, Oxford, UK.
6. Population Health Innovation Lab, Department of Public Health, Environments & Society, Faculty of Public Health & Policy, London School of Hygiene & Tropical Medicine, London, UK.
7. Department of Urban Planning, College of Architecture and Urban Planning, Tongji University, Shanghai, China.
8. Great Ormond Street Institute of Child Health, University College London, London, UK.
9. Global Food System and Policy Research, School of Global Health, Faculty of Health, York University, Toronto, ON, Canada.
10. School of Health and Social Work, University of Hertfordshire, Hatfield, UK.

***Correspondence to:** Matthew Keeble; Email: matthew.keeble@uantwerpen.be

Citation: Keeble M, Chang M, Derbyshire D, et al. Retailer responses to public consultations on the adoption of takeaway management zones around schools: a longitudinal qualitative analysis. Int J Health Policy Manag. 2024;13:8294. doi:10.34172/ijhpm.8294

Supplementary file 1

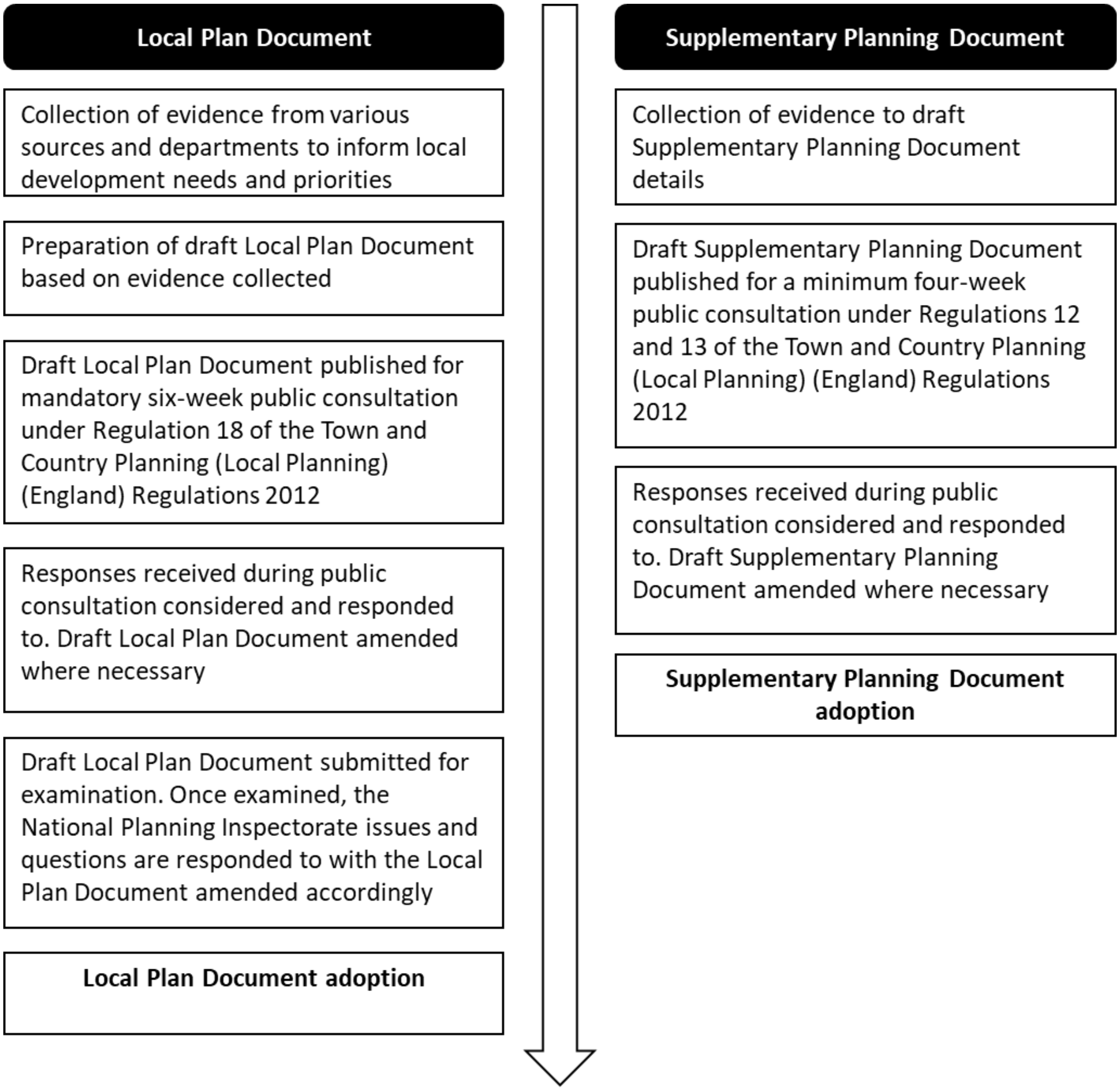

Figure S1. Overview of mandatory public consultation process for Local Plan Documents and Supplementary Planning Documents (adapted from Elmbridge Borough Council).

Box S1. Local authorities in England that adopted a takeaway management zone around schools between 2009 and 2019, included in analysis (n=41).

LOCAL AUTHORITY	YEAR OF ADOPTION	REGION IN ENGLAND
Waltham Forest	2009	London
Barking and Dagenham	2010	London
Rossendale	2011	North West
St Helens	2011	North West
Stockport	2011	North West

Barnsley	2012	Yorkshire and the Humber
Halton	2012	North West
Sandwell	2012	West Midlands
Bolton	2013	North West
Ealing	2013	London
Islington	2013	London
Bradford	2014	Yorkshire and the Humber
Bristol	2014	South West
Enfield	2014	London
Lewisham	2014	London
Medway	2014	South East
Salford	2014	North West
Warrington	2014	North West
Gateshead	2015	North East
Hackney	2015	London
Lambeth	2015	London
Preston	2015	North West
Rochdale	2015	North West
Wandsworth	2015	London
Blackburn with Darwen	2016	North West
Brent	2016	London
Newcastle upon Tyne	2016	North East
Bromsgrove	2017	West Midlands
Kingston upon Hull	2017	Yorkshire and the Humber
Manchester	2017	North West
North Tyneside	2017	North East
Sefton	2017	North West
South Tyneside	2017	North East
Torbay	2017	South West
Wakefield	2017	Yorkshire and the Humber
Hyndburn	2018	North West
Redbridge	2018	London
Richmond	2018	London
Wolverhampton	2018	West Midlands
Coventry	2019	West Midlands
Leeds	2019	Yorkshire and the Humber

Table S1. Theme and code map developed during longitudinal thematic analysis

Theme	Codes	Description and/or elements
The role of takeaways in obesity		
	Broader determinants of obesity	Emphasising that there are numerous and broad determinants of obesity, including broader dietary practices and exercise
	Individual responsibility	Reference to individuals and the way that their lifestyles contribute to them living with obesity
	Takeaways **should** be the focus of planning policies and/or guidance<br><br>Note: data within this code is from local authority responses only	Referring to the ‘problems’ that takeaways create or contribute to, and therefore, aspects that the exclusion zone (or the document that the exclusion zone is detailed in – i.e. an SPD or a Local Plan) might ‘address’ or ‘solve’
	Other sources of unhealthy food	Referring to other retailers that sell unhealthy food
Takeaway management zone adoption		
	Retailers describing local authority policies	Describing how the intervention is an attempt to restrict choice and the freedom (rather than as a way to improve health)
	Non-compliance	Reference to how the proposed policy does not align or conform with the sequential test, the planning framework, or planning strategy and guidance documents
	Multidisciplinary and multifaceted solution<br><br>Note: data within this code is from local authority responses only	Reference to how the proposed solution is part of broader attempts to improve public health that encompass multiple teams and approaches
	Local authorities defending their proposals<br><br>Note: data within this code is from local authority responses only	Local authority responses that defend the concept or details or their proposal
	Alternative non-solutions	Proposal of alternative solutions that promote freedom of choice and that minimise possible economic or commercial impact

	Retailer demands	Proposed amendments to the exclusion zone to fit retailer needs
	Undermining statements	Reasons that the proposed exclusion will fail to be successful
	Unintended or unanticipated consequences	Reference to how the exclusion zone will have systemic impacts that have not been considered and cannot be planned for
Use and interpretation of evidence		
	Favourable selection and interpretation	Consistently selecting and quoting from a limited range of academic research articles and planning guidance documents – essentially cherry-picking which articles and elements of these to refer to<br><br>Note: the scale of cherry-picking is only evident when considering the data as a whole
	Strength of evidence	Reference to hierarchy of evidence from academic research (non-causal and crosssectional) and how evidence did not support the proposed exclusion zone
	Multiple levels and sources	Reference to evidence generated from different levels and sources:<br><br>National – planning guidance (and its apparent non-consideration for diet or health)<br><br>Local – evidence possibly generated from local authorities to support planning policy adoption or reference to planning decisions from inspectors when local authorities previously attempted to adopt and/or implement an exclusion zone (notably, retailers cherry-picked this evidence)<br><br>Reference to difference sources of evidence within these levels
Managing external opinions		
	Foodwashing	Reference to corporate social responsibility activities, including, for example, contributions to employment opportunities, the economy, changing menu items, broader public health action
	Local authority defensiveness	Local authority responses that are defensive of their approach or challenge retailers<br><br>Note: language is particularly important for this code

	Note: data within this code is from local authority responses only	
	Self-image and image of each other	Attempts from retailers and local authorities to influence how they are seen by others, and how others see the retailer or local authority (respectively)
